# Jacques-Louis David (1748–1825). Coronation of Empress Josephine by Napoleon I at Notre Dame de Paris, 2 December 1804 (1806–1807)

**DOI:** 10.3201/eid0910.AC0910

**Published:** 2003-10

**Authors:** Polyxeni Potter

**Affiliations:** * Centers for Disease Control and Prevention, Atlanta, Georgia, USA

**Figure:**
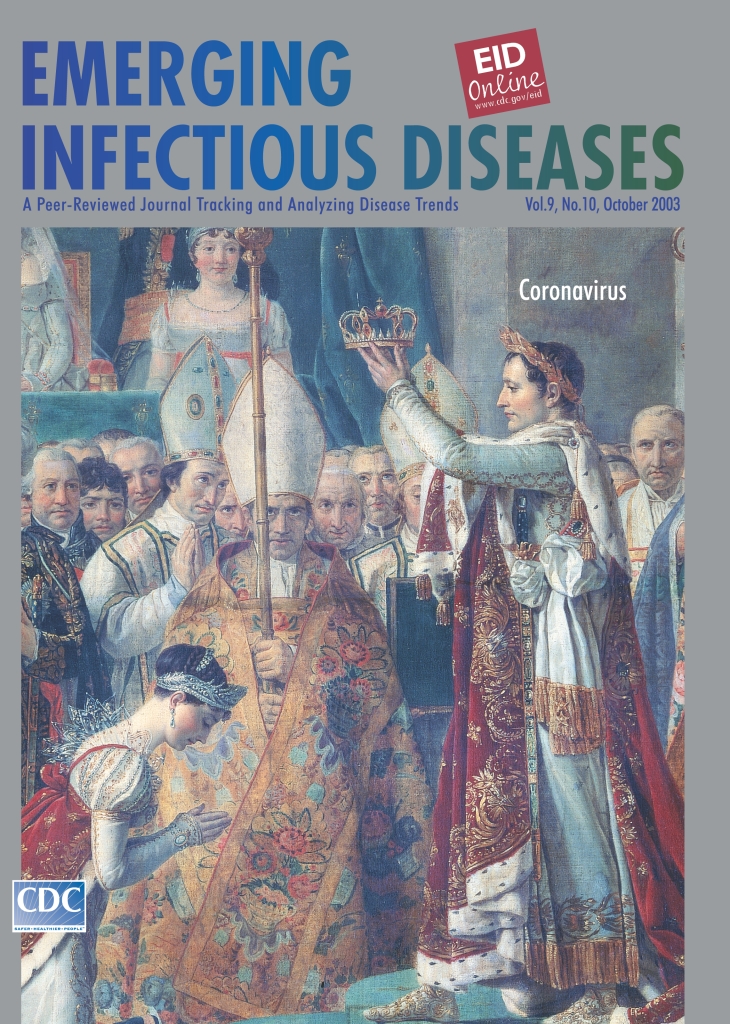
Jacques-Louis David (1748–1825). Coronation of Empress Josephine by Napoleon I at Notre Dame de Paris, 2 December 1804 (1806–1807). Detail of Napoleon and Josephine. Oil on canvas, 6.1 m x 9.31 m. Photo: Peter Willi. Réunion des Musées Nationaux/Art Resource, NY. Chateaux de Versailles et de Trianon, Versailles, France

“I was always hiding behind the instructor’s chair, drawing for the duration of the class,” admitted Jacques-Louis David, acknowledging his early artistic bent (*1*). The orphaned son of a wealthy Paris family, David went on to study art, first under distant relative François Boucher, then under gifted teacher and rococo painter Joseph-Marie Vien. Later, in Italy for 5 years, David became engrossed in archaeology, classical architecture, and mythology, which, along with the paintings of his compatriot Nicolas Poussin, provided inspiration for his work as leading neoclassical painter (*2*).

David’s era was the Age of Enlightenment, whose standard-bearers (David Hume, Voltaire, Jean-Jacques Rousseau, Heinrich Heine, and others) revolutionized economics, politics, and religion, steering them away from authoritarian tradition, toward reason and the common good. The arts, abandoning the baroque, relinquished the ornate, aristocratic, and frivolous excesses of rococo. They turned toward nature and heroic morality, and by extension, toward the ancient “apostles of reason,” the classics, and their “noble simplicity and calm grandeur.” Italy’s artistic leadership declined, leaving the role of guardian of Western art to France and the students of Vien (*3*).

The great political upheavals of the mid-18th century, the American Revolution and the French Revolution, followed the sweeping changes in the world of ideas and ushered in the modern era. David enthusiastically took part in the French Revolution and interpreted the issues of his day in masterpieces drawn from ancient moral dilemmas (The Death of Socrates) and contemporary events (The Death of Marat).

Napoleon Bonaparte rose to power after a coup d’état in 1799. A plebiscite in 1802 confirmed his lifetime rule as Consul of France, in preparation for his becoming Emperor of the French Republic. In 1804, in an elaborate ceremony reminiscent of the coronation of Holy Roman Emperor Charlemagne and in the presence of Pope Pius VII, Napoleon grasped his sword to his heart and put the crown on his own head (*4*). David, then official painter to the emperor, was tasked with commemorating the coronation festivities at the cathedral of Notre Dame. David’s rendition of the event, on this month’s cover of Emerging Infectious Diseases, does not dwell on Napoleon’s imperial indiscretion. Rather, it portrays the crowning, by the emperor, of his wife Josephine. Josephine’s coronation itself is a small part of the massive composition, an enormous group portrait of more than 100 figures (*5*).

Art has been a powerful instrument of revolutions, and David used it often to portray Napoleon as legendary opponent of absolutism, embodying the quest for truth and liberty. Music has similarly served popular uprisings. Beethoven’s Third Symphony, “Eroica,” for a brief time referred to as the Bonaparte Symphony, was inspired by Napoleon’s heroic promise. This epic symphony, which dramatically captures the spirit of humanity, parallels the complexity of revolutionary passions (*6*).

In the eyes of the world, as in the eyes of those attending the lavish coronation in David’s painting, Napoleon’s fall came the moment he assumed imperial status. The laurel leaf crown of Roman emperors was no simple corona. To the champions of equality it symbolized tyranny. David’s political entanglement with the Napoleonic era ended in imprisonment and exile. The dedication to Napoleon in Beethoven’s score of the “Eroica” was retracted. And Josephine was banished to make room for Napoleon’s true mistress, power.

The crown and its elusive promise have downed many a revolutionary hero, the corona of power often becoming halo of disaster. The same is true at times in nature. Some biologic agents are reminiscent of the sun, whose corona is only visible during a full eclipse. The coronaviruses, named for their crownlike appearance in which a loosely wound center is neatly surrounded by club-shaped peplomers, are a case in point. Known animal pathogens for many years, the more than 15 species of coronaviruses infected a variety of mammals and birds yet remained largely obscure, until antigenic variation or some other, unknown, cause brought them into the spotlight. An animal pathogen causing zoonotic infection in humans or a recombinant of human coronavirus and animal virus, severe acute respiratory syndrome virus has brought on a halo of disaster, circling the globe with illness and death.
